# Supplementary material for: Decontamination of Geobacillus Stearothermophilus using the Arca Aerosolized Hydrogen Peroxide decontamination system
Source: PLoS One. 2022 Sep 20;17(9):e0273937. doi: 10.1371/journal.pone.0273937 (PMC9488756; doi:10.1371/journal.pone.0273937)
Supplement: Attachment — Submitted filename: Response to Reviewers.pdf [file pone.0273937.s001.pdf]

To Whom It May Concern:

We would like to express our thanks to the reviewers for their time and commentary. We appreciate your efforts to improve the rigor of our manuscript as well as your dedication to scientific literature at large.

The critiques from both reviewers overlapped in several of their comments, demonstrating that our manuscript lacked some clarity regarding both the Arca's cost-effectiveness as well as our methods to demonstrate sterility.

We have attempted to address these deficiencies in our text. The suggestions and clarification questions provided by the reviewers have improved our paper considerably. Please see our full responses in red to the specific comments below.

Reviewer #1: The manuscript by Mead et al "Decontamination of Geobacillus Stearothermophilus using the Arca Aerosolized Hydrogen Peroxide decontamination system" is a well written description of a device that uses vaporized hydrogen peroxide to disinfect N95 respirators. The methods are straightforward and are consistent with previous literature that vaporized H2O2 is an effective disinfectant.
The main issue to consider is that the authors repeatedly discuss this as an option for low resource areas at a cost of 2000. It is not clear from figure 2 how many masks fit in the device and what the throughput is. In table 1 many of those were high throughput set ups such that it is not an equitable comparison. Perhaps including the masks in Figure 2 would be helpful. A cost per N-95 might be more applicable as if one can only repurpose 20 per day even at 2000 it may not be cost effective especially as manufacturing and stockpiles have increased. It would be helpful in the discussion to discuss throughput (how many masks per day for a single unit) as well as cost in this context.
The comment from Reviewer #1 regarding equitable comparisons of the devices is well-taken. The throughput rates for the commercially available systems are much larger than that of the Arca. However, while the marginal cost to clean an additional N95 is significantly lower for the large throughput commercially available options, the outsized cost of these solutions make them a non-starter for low-resource settings. Additionally, Abaton is a non-profit which provides the Arca devices for free to at-need institutions. The true costs to end users include the purchase of additional H2O2, electricity to run the Arca, and staffing. These are variable costs based on location and utility pricing. We have highlighted this point and included the throughput of the Arca in our updated discussion to better address the cost-effectiveness of the device.

Minor point
The authors state sterility was demonstrated by clear TSB and lack of color change at 7 days. Unfortunately I was unable to access the cited reference (error code) for the steris procedure and the manufacturer of the BI strip is not provided. TSB turbidity requires significant growth and lack of turbidity is not equivalent to no growth. The media will not necessarily change color with small amounts of growth (perhaps this was a reference to a color indicator on the strip) Filtering

the media and plating the filter to catch any organisms is one mechanism to capture small amount of organisms in a volume too large to plate. Some clarification of this procedure as well as the limit of detection for colony forming units would be helpful to the reader. It would also be helpful to note how much growth was seen with the positive control as well to confirm the log killing and disinfection achieved if we assume the TSB was in fact sterile.
This is a valid critique and therefore we have added to our discussion regarding the limitations of this study. The TSB growth medium testing provides a binary outcome of growth versus no growth. STERIS labs were unable to provide log kill information.

Reviewer #2: A very straightforward paper. Only a few minor comments.

1) l 42 - In light of ramping up a variety of responses to the pandemic that drove up supplies of PPE and drove down costs, might be helpful to give an estimate of the times/costs to process 1, 10, 100 etc M-95s would be helpful.
We have added the throughput of the Arca in our discussion to better evaluate the cost-effectiveness of the device.

2) l-90 . Presumably these are G. stearothermophilus spores? Are these BIs the ones used for H2O2 sterilizers, as I believe these spores are not generally the ones most resistant to H2O2, although are for wet heat.
That is correct, we used G. stearothermophilus spores. According to CDC recommendations, G. stearothermophilus is the appropriate biologic indicator for assessing sterilization with H2O2 devices. Please see the link below:
https://www.cdc.gov/infectioncontrol/guidelines/disinfection/sterilization/sterilizing-practices.html

3) Since the TSB incubated with H2O2-treated BIs gave no obvious turbidity/color change, most spores have been killed. But what are the limits of this assessment? I could not access reference 23 to get any information about this, and thus it is not clear what the confidence in "sterility is.
This is a valid critique and therefore we have added to our discussion regarding the limitations of this study. The TSB growth medium testing provides a binary outcome of growth versus no growth. STERIS labs were unable to provide log kill information.

Thank you,

--
Loren Mead, MD
President,
Abaton 501(c)(3)
Protecting frontline health workers with PPE

(c) 484-645-7113
lmead@abaton.care
Facebook | Twitter | Instagram
